# Folate metabolism in myelofibrosis: a missing key?

**DOI:** 10.1007/s00277-024-06176-y

**Published:** 2025-01-23

**Authors:** Giacomo Maria Cerreto, Giulia Pozzi, Samuele Cortellazzi, Livia Micaela Pasini, Orsola Di Martino, Prisco Mirandola, Cecilia Carubbi, Marco Vitale, Elena Masselli

**Affiliations:** 1 Department of Medicine and Surgery, Anatomy Unit, University of Parma, Via Gramsci 14, Parma, 43126 Italy; 2 Hematology and BMT Unit, Parma University Hospital (AOUPR), Via Gramsci 14, 43126 Parma, Italy; 3 Faculty of Medicine, Vita-Salute University-San Raffaele, Via Olgettina 58, Milan, 20132 Italy

**Keywords:** Myelofibrosis, Myeloproliferative neoplasms, Chronic inflammation, Folic acid, Folate receptor, One-carbon metabolism

## Abstract

Folates serve as key enzyme cofactors in several biological processes. Folic acid supplementation is a cornerstone practice but may have a “dark side”. Indeed, the accumulation of circulating unmetabolized folic acid (UMFA) has been associated with various chronic inflammatory conditions, including cancer. Additionally, by engaging specific folate receptors, folates can directly stimulate cancer cells and modulate the expression of genes coding for pro-inflammatory and pro-fibrotic cytokines.

This evidence could be extremely relevant for myelofibrosis (MF), a chronic myeloproliferative neoplasm typified by the unique combination of clonal proliferation, chronic inflammation, and progressive bone marrow fibrosis. Folate supplementation is frequently associated with conventional or investigational drugs in the treatment of MF-related anemia to tackle ineffective erythropoiesis. In this review, we cover the different aspects of folate metabolism entailed in the behavior and function of normal and malignant hematopoietic cells and discuss the potential implications on the biology of myelofibrosis.

## Introduction

Myelofibrosis is a stem cell-derived clonal hematological malignancy, operationally classified among the classical Philadelphia-negative chronic myeloproliferative neoplasms (MPN). MF is the prototype of onco-inflammatory disorders and consists of two entities: primary MF (PMF) and post-polycythemia vera (PPV)/post-essential thrombocythemia (PET) MF, also known as secondary MF (sMF). Perturbation of the JAK/STAT signaling pathway is the hallmark of MF (and MPN in general), which provides a selective advantage to the neoplastic clone over normal hematopoietic stem cells (HSCs) and elicits a myeloproliferative phenotype [1].

Malignant hematopoietic stem/progenitor cells are also the main source of a plethora of pro-inflammatory cytokines, reactive oxygen species, and growth factors capable of perturbing tissue homeostasis at both local (bone marrow, BM) and systemic levels, leading to chronic pro-inflammatory state [2, 3]. In the BM, activated stromal cells produce reticulin and collagen fibers, resulting in bone marrow fibrosis (BMF). Higher degrees of BM fibrosis are associated with a more severe disease stage with a dismal prognosis and a higher risk of leukemic evolution [4].

The chronic pro-inflammatory state and the progressive disruption of BM architecture play a major role in the onset of ineffective erythropoiesis, which underlies MF-related anemia. Prevalence of anemia increases with the duration of the disease: from 35 to 38% at the time of diagnosis, scaling up to 58% within 1 year and 64% beyond 1 year from diagnosis, and it has been associated with poor quality of life and reduced overall survival [5]. Current treatments (red blood cell transfusions, erythropoiesis-stimulating agents, androgens, steroids, and immunomodulatory drugs) are associated with multiple side effects and have limited efficacy and durability of response. Encouraging results are emerging from novel therapies, including luspatercept, new-generation JAK-inhibitors (momelotinib, pacritinib), pelabresib (a bromodomain extra-terminal domain inhibitor), imetelstat (a telomerase inhibitor), and navitoclax (a BCL-2/BCL-xL inhibitor) [5, 6].

However, anemia still accounts for one of the most relevant clinical challenges in MF patients. Folate supplementation is often coupled in routine practice with the above-mentioned treatments to tackle ineffective erythropoiesis.

Nevertheless, folates have multiple, crucial biological functions as enzyme cofactors involved in nucleotide synthesis during cell proliferation, epigenetic regulation, and redox balance. Folate over-intake may lead to the accumulation of unmetabolized folic acid that has been associated with several chronic inflammatory conditions, including cancer [7]. Folate can modulate the expression of genes encoding for pro-inflammatory and pro-fibrotic cytokines, and, eventually, stimulate cancer cells directly by engaging specific folate receptors.

In this review, we will discuss the different aspects of folate metabolism with potential implications in the biology of myeloid malignancies, in particular myelofibrosis.

## Folate-mediated one-carbon metabolism (FOCM) and its relevance in cancer

Folates are water-soluble vitamins sharing a fundamental core structure composed of three chemical moieties: a pteridine ring that can undergo reduction or oxidation, a para-aminobenzoic acid (PABA) linker, and a polyglutamate tail. The pteridine ring and PABA linker bind one-carbon (1 C) units, while the polyglutamate tail anchors the molecule inside the cell [8]. Folates serve as a group of enzyme cofactors that allow the transfer of 1 C units for essential cellular processes, such as purine and pyrimidine biosynthesis, amino acid homeostasis, epigenetic maintenance, and redox defense. They require active systems for cellular uptake. Several genetically distinct and functionally diverse transport systems have been identified: the reduced folate carrier (RFC), the proton-coupled folate transporter (PCFT), and folate receptors (FR) [9]. RFC is the main transporter for reduced folate uptake in various tissues at physiological pH; whereas PCFT is responsible for intestinal folate absorption in the acidic pH of the upper small intestine. The folate uptake mediated by FR occurs through internalization of the ligand-receptor complex by endocytosis. Once internalized, the endosome fuses with lysosomes to release folates in the cytosol [10]. Alternatively, folate binding to FRs can trigger downstream signaling pathways such as JAK/STAT and ERK1/2 signaling, as described below.

**Fig. 1:**
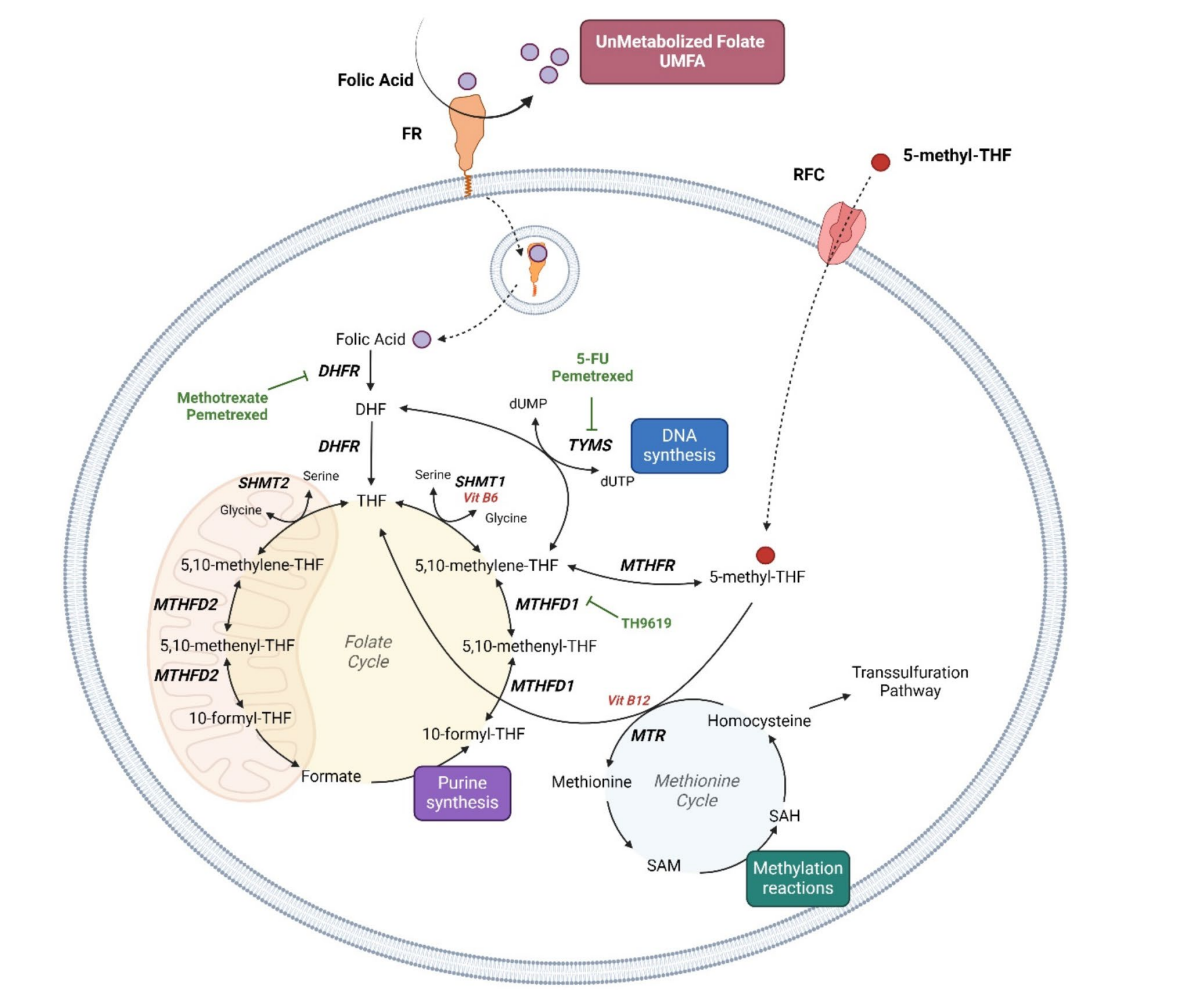
Overview of Folate-mediated One-Carbon Metabolism (FOCM) and crucial steps targeted by anti-cancer drugs. Once internalized by FR and RCF, folic acid (FA) goes through a series of redox enzymatic reactions within cells, known as FOCM. These reactions involve the subsequential conversion of 1 C units into various oxidation states by both cytosolic (SHMT1 and MTHFD1) and mitochondrial (SHMT2 and MTHFD2) enzymes. The products of FOCM, including 10-formyl-THF, 5,10-methylene-THF and 5-methyl-THF are crucial for DNA and purine synthesis, as well as methylation reactions. 5-methylTHF enters the cell through RFC and serves as a substrate for the MTR and vitamin B12-mediated re-methylation of homocysteine to methionine. This reaction also produces THF, replenishing the starting substrate of FOCM. Excessive FA can overwhelm the internalization capacity of folate transporter systems leading to increased levels of circulating UMFA. *Created in BioRender (BioRender.com/w80m96). Abbreviations: FR*,* folate receptor; RFC*,* reduced folate carrier; FA*,* folic acid*,* UMFA*,* unmetabolized folate; FOCM*,* folate-mediated one-carbon metabolism; DHF*,* dihydrofolate; DHFR*,* dihydrofolate reductase; THF*,* tetrahydrofolate; SHMT1/2*,* serine hydroxymethyltransferase 1/2; MTHFD 1/2 methylenetetrahydrofolate dehydrogenase–cyclohydrolase 1/2; MTHFR*,* methylene tetrahydrofolate reductase; 5-FU*,* 5-fluoruracil*,* dUMP*,* deoxyuridine monophosphate; dTMP*,* deoxythymidine monophosphate*,* TYMS*,* thymidylate synthase; MTR*,* methionine synthase; SAM*,* S-adenosylmethionine; SAH*,* S-adenosylhomocysteine*

The folate metabolism, known as **folate-mediated one-carbon metabolism (FOCM)**, represents a network of interconnected reactions that occur in mitochondria and cytosol, simultaneously [11]. Crucial steps of FOCM are reported in Fig. 1. FOCM is essential for the supply of nucleotides for DNA synthesis and repair, according to the proliferative cell demands. In cancer cells, characterized by rapid and unleashed proliferation, enzymes involved in the 1 C metabolism pathway, as the mitochondrial serine hydroxymethyltransferase, SHMT2, and the methylenetetrahydrofolate dehydrogenase MTHFD2, are commonly upregulated to produce the required nucleotides and amino acids [12, 13].

Of the two, MTHFD2 is one of the most highly upregulated genes in cancer, including breast cancer and colorectal cancer [14].

MTHFD2 overexpression boosts folate cycle activity in the mitochondria, generating the formate overflow that fuels the nucleotide synthesis needs of cancer cells [14]. Notably, a MTHFD2 mitochondrial isoform specific to the hematopoietic lineage has been described. MTHFD2 plays a role in normal hematopoiesis, as demonstrated by the evidence that MTHFD2-KO mice embryos fail liver hematopoiesis and show growth defects [14].

In malignant hematopoiesis, Pikman et al. demonstrated that the knockdown of MTHFD2 in acute myeloid leukemia (AML) cells restrained cell growth, colony formation, and promoted differentiation, resulting in decreased leukemia burden and prolonged survival of AML mouse models [15].

MTHFD2 shapes therefore as a potential target for anticancer drugs. Indeed, in their pioneering study, Green et al. described the mechanisms of a novel antifolate, TH9619, which causes thymineless death of MTHFD2-expressing cancer cells in vitro. TH9619 inhibits MTHFD1 in the cytosol, preventing formate-derived 10-formyl-THF conversion into 5,10-meTHF, leading to 10-formyl-THF accumulation and blockage of THF regeneration. At the same time, THF consumption in thymidylate synthesis further contributes to THF depletion, leading to the so called “toxic folate trapping”. This folate trap relies on physiologic hypoxanthine levels as these prevent the activation of the de novo purine synthesis pathway and the use of 10-formylTHF.

Interestingly, in the MTHFD2^KO^ model lacking formate overflow, the same agent displayed cytostatic effects due to purine deficiency [16].

FOCM products (specifically 5-methyl-THF ) also serve as enzyme cofactors for the synthesis of methyl-carrier precursors such as methionine, which, upon conversion into S-adenosylmethionine (SAM), functions as a “universal methyl donor” for > 200 methyltransferases for methylation reactions involving DNA, RNA, and proteins, playing, therefore, a major role in epigenetics [17].

Methionine availability impacts the ratio of SAM to S-adenosylhomocysteine (SAH), which, in turn, affects many methylation reactions including histone methylation.

SAM/SAH ratio is tightly regulated by mechanisms such as (i) MTHFR (methylene tetrahydrofolate reductase) feedback inhibition by SAM [18], (ii) inhibition of the SAM-consuming enzyme glycine-N-methyltransferase by 5-MTHF, (iii) serine hydroxy methyltransferase mediated cytosolic sequestration of 5-MTHF. SAM/SAH ratio imbalance therefore leads to epigenetic dysregulation and cancer promotion. SAM/SAH ratio reduction caused by either folate starvation, polymorphism-associated reduction of MTHFR activity, or SAH administration, was indeed shown to suppress H3K27 and H3K9 methyltransferases and target methylation across the whole genome of AML cells, mediating resistance to MYC-targeting therapies [19].

## Is FA administration a harmless routine practice? Potential implications for myelofibrosis

### UnMetabolized folic acid (UMFA): the “dark side” of folates

Natural folates are found in foods (mainly leafy green vegetables) with 5-methyl-tetrahydrofolate (5-MTHF) being the predominant form. Folic acid (FA), or pteroylglutamic acid, is a synthetic folate and the most found vitamin in fortified foods and over-the-counter supplements.

Periconceptional FA supplementation (400 ug daily) is a cornerstone practice for reducing the risk of neural tube defects (NTDs). Since 1998, cereal-grain product fortification (40 µg of FA per 100 g of product) has been mandated first in the US and then in other 80 countries worldwide to increase the FA intake of all women of childbearing age to decrease birth-related NTDs [7].

Other than in pregnancy, FA supplementation is also indicated for other medical conditions, i.e. folate-deficiency anemia (5–15 mg daily) [20], dietary deficiency, or chronic hemodialysis (1–5 mg daily) [21]. FA supplementation is also a common clinical practice in autoimmune and non-immune hemolytic anemias (such as sickle cell disease) and hemoglobinopathies characterized by ineffective erythropoiesis (such as beta-thalassemia), although robust evidence of a clear benefit in these conditions is lacking [22, 23].

Due to fortification measures and the widespread use of supplements, individuals might be exposed to FA levels surpassing the recommended intake levels, i.e. 0.33–0.40 mg of dietary folate equivalents in 1500 kcal, or 0.40 mg, according respectively to ESPEN and the Institute of Medicine [24].

Indeed, the current average U.S. daily intake, as dietary folate equivalent, is 0.70 mg, of which 0.20 mg occurs naturally in food and 0.50 mg derive from fortification or other sources, i.e. supplements [7]. Individuals getting FA from supplements are also at risk of surpassing the upper tolerable intake level of folates, which has been set to 1.00 mg by both the Institute of Medicine and the European Food Safety Agency, based on the risk of developing a masked cobalamin deficiency [24, 25].

Whilst folate excess may be relatively safe in healthy conditions, there are concerns that it may become detrimental during chronic inflammatory conditions, including cancer. Indeed, FA intakes over 0.20 mg/die, or 0.40 mg/die– according to different authors - may lead to an increase in circulating **UnMetabolized Folic Acid (UMFA)** [7]. While the naturally occurring dietary 5-MTHF directly enters cellular processes, FA must undergo several sequential enzymatic reactions, catalyzed by the enzyme dihydrofolate reductase (DHFR), before entering the folate cycle as THF (Fig. 1). In humans, hepatic DHFR exhibits very weak activity, and therefore doses of dietary FA easily saturate DHFR, resulting in UMFA entering the circulation [7].

The role of excess folic acid intake, elevated folate status, and UMFA in disease is extremely controversial [26]. Elevated folate status has been associated with an increased risk of various disease conditions, including cancer. Effects seem more consistent and univocal on prostate cancer (O.R. up to 5.82), hepatocellular carcinoma (HR 2.05), and lung cancer (H.R. 1.59), whilst conflicting data are available on colorectal and lung cancer [27, 28]. The most robust evidence derived from combined analyses and extended follow-up of two vitamin B intervention trials among patients with ischemic heart disease in Norway (where there is no folic acid fortification), documenting that treatment with 0.8 mg/d of folic acid was associated with increased cancer incidence, cancer mortality, and all-cause mortality [27]. Remarkably, in this cohort, estimated HRs for hematologic cancer incidence and hematologic cancer mortality in the folic acid vs. non-folic acid groups were 1.43 (0.70–2.93 95% CI) and 1.75 (0.51–5.94, 95% CI), respectively [27]. On the other side, a comprehensive metanalysis on the effects of FA supplementation on overall and site-specific cancer incidence during the randomized trials fail to demonstrate an association between FA supplementation and increased incidence of site-specific cancer during the first 5 years of treatment [29].

A UMFA/fibrosis nexus is also emerging, as indicated by evidence of long-term fibrogenic changes induced by higher concentrations of folic acid in the kidney and liver [30, 31].

Intriguingly, Quinn et al.. identified soluble secreted folate receptor γ (FRγ or FOLR3) as a pivotal player in human metabolic dysfunction–associated steatohepatitis (MASH). FOLR3 is highly upregulated in human MASH (metabolic dysfunction-associated steatohepatitis) livers, and its levels correlated with the disease stage. Hepatic stellate cells ex-vivo stimulation with FOLR3 increases extracellular matrix production by blocking HTRA1-mediated negative regulation of transforming growth factor β 1 (TGFβ1), a master regulator cytokine of fibrosis [32].

Notably, as discussed below, TGFβ1 plays a crucial role in the pathobiology of myelofibrosis by promoting BM fibrosis and collagen deposition and by enhancing the dormancy of normal HSCs [33].

This evidence warrants further investigation to uncover a potential role of FOLR3 in enhancing fibrosis in MPN.

### Indirect effects of FA on pro-inflammatory cytokine/receptor axes

Being fibrosis a key physiologic mechanism of wound healing, its basic mechanisms overlap among different organ systems [7, 34] some of these being: (i) the involvement of cytokine-mediated mesenchymal and immune cell recruitment, (ii) mesenchymal stem cell differentiation to myofibroblasts, (iii) the secretion and accumulation of extracellular matrix proteins, i.e. collagen, leading to the subversion of tissue morphology and functionality.

The most relevant chemokine/cytokine axes reviewed in Pozzi et al. [35] recently described as pathophysiologically relevant in MF fibrosis are:

- **TGFβ-mediated signaling**. TGFβ is a pro-fibrogenic mediator produced by the MF megakaryocytic (MK) clone, that does not express the hematopoietic transcription factor GATA1 and is tagged by typical morphological abnormalities and peculiar transcriptional profile enriched in pro-inflammatory pathways [33, 36, 37].
- **IL-1/IL-1R signaling**. IL-1 is a master regulator of inflammation and seems to promote fibrosis acting on both BM mesenchymal stromal cells and the MK clone [38].
- **CCL2/CCR2 signaling**. The expression of the monocyte chemokine receptor CCR2 on MF hematopoietic stem/progenitor cells correlates with the degree of BM fibrosis and the engagement with its ligand CCL2 activates pro-proliferative signals [39, 40].
- **Interleukin (IL)-13/IL-4 signaling.** IL-13 and IL-4 are involved in type II inflammatory response; IL-13 overproduction appears to promote fibrosis prompting the proliferation of the MK clone and the activation of BM mesenchymal stromal cells, while IL-4 blockage ameliorates fibrosis [34].
- **CXCL8/CXCR1/2 axis.** MF hematopoietic stem/progenitor cells from patients with advanced fibrosis are enriched for a CXCL8/CXCR2 gene signature and the expression of the receptor CXCR1/2 correlates with the degree of BM fibrosis [41].

Intriguingly, a growing body of evidence supports an anti-inflammatory effect of FA in some of these pathways, mediated by its role in DNA methylation reactions– as in the case of IL-1β, or by indirectly modulating gene transcription– as in the case of CCL2.

DNA methylation state has been suggested as a regulatory mechanism behind several chronic inflammatory diseases such as spondylarthritis, inflammatory bowel diseases, and psoriasis [42].

Samblas et al. [43] demonstrated that FA can reduce *IL1β* mRNA expression and protein secretion in LPS-stimulated human THP-1 monocyte/macrophage, by increasing the methylation levels in CpGs located in *IL1B* gene.

Moreover, Lu et al. showed that Ea.hy 926 endothelial cells grown in chronic low folate condition (“folate stress”) display increased CCL2 mRNA levels, due to enhanced CCL2 transcription mediated by phospho-p38, suggesting that under chronic folate stress, endothelial cells are “primed” via elevated phospho-p38 levels for increased CCL2 production [44].

### Direct effects of FA on hematopoietic cells mediated by folate receptors

As above described, folates use diverse transport systems to enter the cells. Due to their relevance in cancer, we will focus on FR, a group of glycosylphosphatidylinositol-anchored proteins selectively expressed by tissues that bind FA and 5-MTHF with high affinity [9].

In humans, the following four isoforms have been characterized to date: FRα (*FOLR1*), FRβ (*FOLR2*), FRγ (*FOLR3*), and FRδ (*FOLR4)* [45].

**FRα** and **FRβ** are anchored to the plasma membrane and bind folic acid with the highest affinity, whilst FRγ is secreted by myeloid cells (primarily neutrophils and monocytes), as it lacks the membrane-anchoring glycosylphosphoinositol (GPI) domain [46, 47]. **FRδ** is expressed in regulatory T lymphocytes and mammalian eggs, although exhibiting no affinity for folic acid in these cells [47, 48]. Indeed, the ability to bind folic-like molecules appears to be an exclusive characteristic of tumor-associated Treg [49].

Expression and function of different FR isoform in normal conditions and disease are summarized in Table 1.

Expression of FRα is restricted to the luminal surface of polarized epithelia of organs such as the kidney, lung, choroid plexus, and placenta [50, 51], whilst FRβ is expressed by myeloid-lineage cells, placenta, spleen, and thymus [52, 53]. In the normal development of the myelomonocytic lineage, FRβ, along with CD14, is considered a differentiation marker at relatively low levels in monocytes [54] (Table 1).

Notably, FRα is aberrantly expressed in epithelium-derived cancers (ovarian, kidney, lung, brain, endometrial, colorectal, pancreatic, gastric, prostate, testicular, bladder, head, and neck, and breast cancer), and it has been linked to neoplastic progression and poor prognosis in a subset of them [55, 56] (Table 1). Recent evidence indicates that FRα activates intracellular signaling pathways not related to 1 C metabolism. Notably, FRα mediates JAK–STAT3 and ERK1/2 signaling pathway activation [9, 57] (which, as mentioned above, are key molecular events in MPN). Given the high sequence identity (82%) and similarity (92%) among FRα and FRβ [54], these mechanisms may be shared also by FRβ.

**Table 1:** Distribution of FR isoforms among tissues in normal and pathological conditions

Receptor	Cellular localization	Capacity to bind folate	Tissue distribution	Activated Signaling Pathways	References
FRα	Membrane	+	*Normal tissue/cells* Kidney, lung, choroid plexus, and placenta epithelia		[50, 51]
		++	*Pathological Tissues/Cells* Ovarian, kidney, lung, brain, endometrial, colorectal, pancreatic, gastric, prostate, testicular, bladder, head and neck, and breast cancer. **AMKL blasts**	JAK/STAT3 signaling ERK1/2 signaling	[55, 56] [57] [9] [75]
FRβ	Membrane	+ **+/-** + + +	*Normal tissue/cells* Placenta, spleen, and thymus epithelia **CD34 + cells** **Neutrophils** **Classic pro-inflammatory monocytes** **M2-polarized macrophages**		[53] [52, 58] [52] [52, 60, 61] [62, 65]
		+ + ND ++	*Pathological Tissues/Cells* **TAM** in primary and metastatic melanoma; breast cancer and lung cancer **M2** alveolar macrophages in IPF **CML blasts** **AML blasts**	Fibrogenic signaling pathways	[62–66] [69] [52] [62]
FRγ	Soluble	+	*Normal tissue/cells* Released by Neutrophil granulocytes and monocytes		[46] [47]
		+	*Pathological Tissues/Cells* Liver in MASH **CML cells (** ***FOLR3*** **gene expression)**	TGF-β-mediated fibrogenic signaling pathway	[32] [80]
FRδ	Membrane	-	*Normal tissue/cells* Regulatory T cells, mammalian egg cells		[47, 48]
		+	*Pathological Tissues/Cells* Tumor-associated Treg		[49]
Undetermined isoform	ND	ND	*Pathological Tissues/Cells* MF spleen		[78]

AML, Acute Myeloid Leukemia; AMKL, Acute Megakaryoblastic Leukemia; CML, Chronic Myeloid Leukemia; ERK1/2, Extracellular signal-regulated kinase 1/2; IPF, Idiopatic Pulmonary Fibrosis; JAK/STAT3, Janus Kinase/Signal Transducer and Activation of Transcription 3; MASH, Metabolic Dysfunction–Associated Steatohepatitis; MF, myelofibrosis; ND, not determined; TAM, tumor-associated macrophages; TGF-β, Transforming Growth Factor-Beta; Treg, regulatory T cells In bold are highlighted hematopoietic cells

#### FR expression in normal hematopoietic stem and myeloid cells

FR expression in normal hematopoietic stem cells is controversial. Reddy et al. in 1999 demonstrated that FRβ and γ, but not FRα, are expressed on human CD34 + cells isolated from BM samples. However, the functional role of this receptor is still unclear, since FR on hematopoietic cells does not bind to FA, and no detectable FA transport has been documented in these cells [58]. Conversely, one year later, Ross et al. showed low or insignificant co-expression of FR-β with CD34 in both peripheral blood and bone marrow hematopoietic stem cells [52]. This latter observation is supported by the absence of hematotoxicity from FRβ targeted therapies in AML, as discussed below [59].

In differentiated myeloid cells, FRβ is surprisingly expressed on both inflammatory monocytes (transient expression) and anti-inflammatory tumor-associated macrophages (Table 1).

According to Samaniego et al., the PU.1 transcription factor, which is preferentially expressed in myeloid cells and controls the expression of the M-CSF receptor, enhances the transcriptional activity of the proximal regulatory region of the *FOLR2* gene and directly influences *FOLR2* gene expression [60].

The classic/proinflammatory CD14^high^CD16^−^CCR2 + monocytes uniquely express FRβ and are capable of binding FA through this receptor. These monocytes are the only FRβ + cells in peripheral blood in physiological conditions (Table 1) [52, 61].

Conversely, FRβ is a marker for M2-polarized macrophages and correlates with their increased folate uptake ability. The expression of FRβ is promoted by M-CSF, maintained by IL-4, and prevented by the key driver cytokines of M1 phenotype: GM-CSF and IFNγ. Consistently, FRβ is detected in tumor-associated macrophages (TAM) that exhibit an M2-like functional profile and potent immunosuppressive functions within the tumor environment (Table 1) [62].

Immunohistochemistry studies revealed that FRβ is frequently co-expressed with CD163 in TAM from primary and metastatic melanoma and in the stroma of lung [63], ovary [62], colon, gastric [64], and breast cancers [65], where numerous CD68^+^ TAM were also present [62] (Table 1).

However, the role of FRβ + TAM in cancer cell behavior is controversial. Indeed, in breast cancer, FRβ + TAM localize in perivascular areas in the tumor stroma, where they interact with CD8^+^ T cells. The density of FRβ + TAM positively correlates with better patient survival, likely via CD8^+^ T cell priming [65]. By contrast, FRβ overexpression in lung cancer TAMs was associated with poor prognosis [66].

The mechanism underlying the expression of FRβ in M2 TAM macrophages has not been ascertained yet; however, it is hypothesized that the proliferation and activation of macrophages and fibroblasts found in these conditions requires a large amount of folate for enhanced cell replication [67].

M2 macrophages can also induce fibroblasts to differentiate into myofibroblasts to promote fibrosis, as has been demonstrated for idiopathic pulmonary fibrosis (IPF).

Interestingly, alveolar macrophages express higher FRβ in IPF patients and are a source of large amounts of TNF and ROS upon inflammatory stimulus [68]. Notably, using recombinant immunotoxin to clear FR-β positive macrophages can reduce pulmonary fibrosis induced by bleomycin [69].

This could be of utmost relevance in MPN, in which the development of BM fibrosis and the differentiation of mesenchymal stromal cells to profibrotic myofibroblasts depend on macrophages. CD68 + and CD163 + macrophage abundance differs in MPN subtypes, with MF having the highest frequency in BM biopsies, followed by PV and ET [70]. Additionally, in MF, the grade of BM fibrosis significantly correlated with the frequency of CD68 + and CD168 + macrophages. Furthermore, treatment with the JAK1/2 inhibitor ruxolitinib results in an improvement of BM fibrosis, a decrease of CD68 + macrophages, and modulation of the CD163 + M2 subtype in approximately half of the patients [71].

#### FR expression in myeloid malignancies

Since in normal hematopoietic cells only FRβ of activated macrophages has been univocally proved to bind folate, its expression on malignant hematopoietic clones may have significant diagnostic and therapeutic implications.

FRβ is present in its functional form in approximately 70% of AML, generally at low and extremely variable levels [72] (Table 1), but its expression can be induced by the combination of all-trans retinoic acid and valproic acid [73].

A potential strategy to selectively target FRβ^+^ AML cells has been pioneeringly described by Lynn et al., developing a high-affinity FRβ-specific chimeric antigen receptor (CAR)-T cells capable of eradicating AML and normal myeloid lineage without HSC toxicity [59]. More recently, Roy and colleagues utilized the *m909* anti-FRβ antibody to promote AML cell ablation via antibody-dependent cellular cytotoxicity in in-vitro and ex-vivo models, without significant toxicities. The antibody was also successfully tested on in-vivo patient-derived xenograft model; however, its exact mechanism in vivo is not yet clear [74].

Notably, FRα, usually not expressed by hematopoietic cells, has been recently pinpointed as a unique marker and potential target in Acute Megakaryoblastic Leukemia (AMKL) [75] (Table 1). *FOLR1* is uniquely expressed in AMKL while it is absent in other AML and in normal bone marrow bulk samples and peripheral blood CD34^+^ cells. FRα is detected by flow cytometry on AMKL blasts but not on normal lymphocytes, monocytes, and myeloid cells within individual patients. Of note, FRβ exhibits limited expression in this subset of patients. FRα has been successfully targeted with CAR-T cells in a patient-derived xenograft murine model of the disease [75].

Data on FR expression in *BCR::ABL1*-positive chronic myeloid leukemia (CML) is scarce (Table 1). A report dating back to early 2000 describes the presence of FRβ on CML cells [52]. More recently, *FOLR3* has been identified as one of the top differentially expressed genes in relapsed (lower expression) vs. non-relapsed (higher expression) patients after tyrosine kinase inhibitor (TKI) cessation. This is likely due to the presence of the rs139130389 SNP, as, FOLR3 SNP + CML cells do exhibit the lowest *BCR-ABL1* expression and are more sensitive to TKI.

Indeed, higher FRγ expression could lead to an increase in folate available to SNP + clonal cells, paradoxically leading to a reduction in MTHFR activity [18, 76] and generating a folate deficiency-like phenotype, favoring oxidative damage and genomic instability [77].

Intriguingly, these same conditions may otherwise be detrimental to therapy success as evidenced by reports of folate starvation and consequent MTHFR activity reduction, driving BET inhibitor resistance in AML [19]. In the same study, MTHFR^KO^ conditions were also evaluated, leading to similar conclusions.

Only one, dated, report is available on FR expression in MF (Table 1), documenting the presence of a folate-binding protein in the spleen lysate of a patient with “agnogenic myeloid metaplasia and myelofibrosis” [78]. We can speculate that this/these folate receptor(s) may be either expressed by M2-polarized macrophages present in the spleen [79] and/or MF stem/progenitor cells.

## Perspectives

Folates are involved in a plethora of essential biological processes, from cell proliferation to epigenetic regulation, and therefore may affect cancer cells in many ways. As emerging from the present review of the literature, our current knowledge of the role of folates in hematologic malignancies is still fragmentary and mainly focused on the expression of FR isoforms on the malignant clone. These data, although providing invaluable information for diagnosis and therapy, in our opinion offer only a partial view of how folates can have an impact on myeloid neoplasms, in particular MF. MF is uniquely typified by the combination of clonal proliferation, chronic inflammation, and progressive fibrosis, with end-stage ineffective hematopoiesis and increased risk of leukemic transformation [1].

**Fig. 2:**
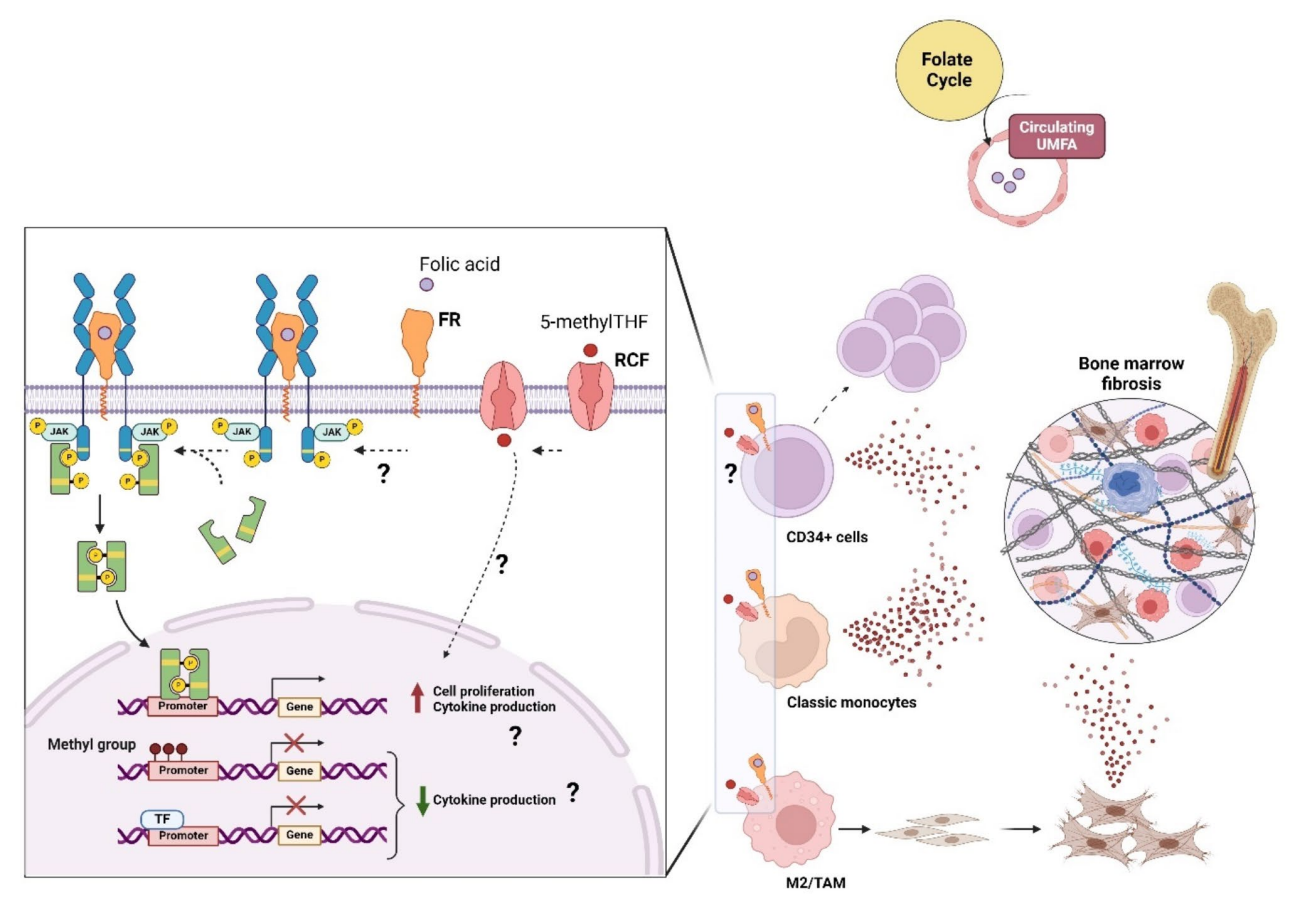
Exploring the potential mechanisms of how folate can affect MF. FA can interact with/enter immune cells expressing FR or RCF. In immune cells it can activate: (i) both pro-inflammatory and anti-inflammatory gene expression programs; (ii) pro-proliferative signals via JAK/STAT3 and ERK1/2 pathways; (iii) M2/TAM-induced fibroblast differentiation in myofibroblasts. All these events may contribute to clonal expansion and bone marrow fibrosis. FA might promote the fitness of the malignant clone directly in case of expression of FR on MF CD34 + cells. Fibrosis can also be promoted by excess folate intake, leading to circulating UMFA. *Created in BioRender (BioRender.com/w80m97)*

Relying on current knowledge of folate metabolism and biological functions, we can speculate that folate may intersect MF pathophysiology and natural disease history at different levels, with even opposite effects (Fig. 2):

- MF stem/progenitor cells, via FR expression.
- MF milieu, via FR expression on M2 macrophages.
- MF fibrosis and systemic inflammation, via UMFA build-up.
- MF systemic inflammation, via down-modulation of cytokine/chemokine expression.

In the first three cases, FA would mediate a detrimental effect, while in the latter case, FA would prompt a beneficial effect of the disease.

Overall, these aspects need to be carefully considered when administering folate to support erythropoiesis in MF-related anemia.

We believe that the evidence discussed in this review warrants the need for further research to dissect this potentially controversial role of folate in MF and provide therefore novel insights into disease pathophysiology and therapy.

## Data Availability

No datasets were generated or analysed during the current study.
